# Safety Evaluation of Chinese Medicine Injections with a Cell Imaging-Based Multiparametric Assay Revealed a Critical Involvement of Mitochondrial Function in Hepatotoxicity

**DOI:** 10.1155/2015/379586

**Published:** 2015-02-22

**Authors:** Meng Wang, Chen-Xiang Liu, Ran-Ran Dong, Shuang He, Ting-Ting Liu, Tie-Chan Zhao, Zhi-Long Wang, Xi-Ya Shen, Bo-Li Zhang, Xiu-Mei Gao, Yan Zhu

**Affiliations:** ^1^Tianjin State Key Laboratory of Modern Chinese Medicine, Tianjin University of Traditional Chinese Medicine, 312 Anshanxi Road, Nankai District, Tianjin 300193, China; ^2^Research and Development Center of TCM, Tianjin International Joint Academy of Biotechnology & Medicine, 220 Dongting Road, TEDA, Tianjin 300457, China; ^3^Molecular Cardiology Research Institute, Tufts Medical Center and Tufts University School of Medicine, 750 Washington Street, Boston, MA 02111, USA

## Abstract

The safety of herbal medicine products has been a widespread concern due to their complex chemical nature and lack of proper evaluation methods. We have adapted a sensitive and reproducible multiparametric cell-based high-content analysis assay to evaluate the hepatic-safety of four Chinese medicine injections and validated it with classical animal-based toxicity assays. Our results suggested that the reported hepatotoxicity by one of the drugs, Fufangkushen injection, could be attributed at least in part to the interference of mitochondrial function in human HepG2 cells by some of its constituents. This method should be useful for both preclinical screen in a drug discovery program and postclinical evaluation of herbal medicine preparations.

## 1. Introduction

Traditional Chinese medicine (TCM) injection is an innovative and quick-acting dosage form of herbal medicine products. It plays a significant role in clinical treatment of acute, severe syndromes in China. However, the safety of TCM injection has been a long concern of the public, especially after consecutive reports of adverse drug reaction (ADR) recently. The ADR induced by TCM injection accounts for 77.2% of all the ADR induced by TCM in national ADR case report database [1]. Hepatotoxicity is a major cause for the termination of drug development programs and frequently results in regulatory actions including denied approval and black box warnings [2]. Drug-induced hepatotoxicity accounts for one-half of cases of acute liver failure in America and Great Britain [3]. Long term and acute animal toxicity test on liver injury was demanded officially in preclinical research. However, classical animal study is inefficient, for only approximately half of new drugs with hepatotoxicity can be found in animal test [4]. A more precise, rapid, and high-throughput method to evaluate the hepatotoxicity of drugs, especially for TCM injection, was needed.

The major mechanistic classifications of hepatotoxicity include inhibition of mitochondrial function, disruption of intracellular calcium homeostasis, activation of apoptosis, oxidative stress, inhibition of specific enzymes or transporters, and formation of reactive metabolites that cause direct toxicity or immunogenicity [5]. HCA is considered as an important predictive tool for application of the above mechanistic understanding for the assessment of hepatotoxicity. It is a recent advance in the automation of quantitative epifluorescence microscopy and image analysis and in the application of microfluorescent, multiprobe technology. It enables kinetic monitoring* in vitro* of cells in real time for multiple cellular biomarkers that are critically involved in the pathogenesis of toxicity [6]. A HCA assay was established to investigate hepatotoxicity of 243 drugs to HepG2 cells. When the data were adjusted to take account of the reported maximum human plasma concentrations of the drugs, a specificity of 98% and a sensitivity of 93% for detection of compounds that cause hepatotoxicity were observed.

TCM injection commonly is a compound preparation without completely clear therapeutic material basis that makes it difficult to evaluate the toxicity effect, especially on its mechanism. The characteristics of visualization, intuition, and multiparameter of HCA are suitable for toxicity assessment of TCM preparations. Four TCM injections, Danhong injection (DHI), Xiangdan injection (XDI), Mailuoning injection (MLNI), and Fufangkushen injection (FFKSI), were selected for the HCA assay. They are all widely used in clinic practice in China with a total sales amount of more than 4 billion RMB in 2013. The hepatotoxicity ADR reports of four injections are varying [7]. XDI and MLNI were reported in ADR information bulletin by SFDA [8, 9]. It was also observed that DHI and FFKSI could increase ALT, AST, and ALP in individual clinical cases [10–14].

The study aimed to develop and validate a practical, reproducible,* in vitro *multiparametric cell-based HCA assay to assess hepatotoxicity of TCM injections and to suggest their mechanisms of action. The assay was applied to HepG2 human cell line [15–17] cultured in 96-well plates. Fluorescent dyes with optical compatibility were adopted to evaluate multiple parameters concerning drug-induced liver injury, including cell number (CN), nuclear area (NA), mitochondrial mass (MS), mitochondrial membrane potential (MMP), and plasma membrane permeability (PMP). Drugs with known hepatotoxic mechanism, P-fluoromethoxyphenylhydrazone (FCCP), acetaminophen, and doxorubicin hydrochloride, were used as positive controls. FCCP, a potent uncoupler of mitochondrial oxidative phosphorylation [18], also reduces MMP activity and induces apoptosis [19]. Acetaminophen is converted by cytochrome P450 enzymes to a reactive metabolite, NAPQI, which was efficiently detoxified by conjugation with GSH. However, after toxic doses, GSH was depleted by the conjugation reaction and the metabolite covalently bounded to protein. Since the loss of GSH, peroxidation reactions occurred by Fenton-type mechanisms which can lead to activation of proteases and endonucleases and DNA strand breaks. The effect of the excess NAPQI on isolated mitochondria and inhibition of mitochondrial respiration are considered to cause damage on hepatocyte [20]. Doxorubicin causes an imbalance between free oxygen radicals (ROS) and antioxidants. The disturbance in oxidant-antioxidant systems results in tissue injury that is demonstrated with lipid peroxidation and protein oxidation in tissue. It is demonstrated that inflammatory processes, free radicals, oxidative stress, and lipid peroxidation are frequently associated with liver damage induced by toxic agents such as doxorubicin [21].

To validate the assay, a 28-day subacute animal study was carried out. Animal mortality, state of survival, body and liver weights, and antioxidant activity in both plasma and liver homogenates were determined and compared with the results of HCA. Hepatotoxicity of the four TCM injections was assessed and the mechanism of action was suggested by the method established.

## 2. Materials and Methods

### 2.1. Drugs and Reagents

FCCP (Sigma, St. Louis, MO, USA, batch number 122M4004V), acetaminophen (Sigma, St. Louis, MO, USA, batch number 061M0042V), and doxorubicin hydrochloride (Meilun Biotech Co., Ltd., Dalian, China, batch number 20120205) were used as positive control to validate the assay. The median concentrations (IC 50) of FCCP and acetaminophen were 5.2 *μ*M and 1.3 mM [22], respectively. The lowest concentration of doxorubicin hydrochloride was 0.1 *μ*M [4]. For dose-response relationship, FCCP at 0.3–30 *μ*M, acetaminophen at 0.3–10 mM, and doxorubicin hydrochloride at 0.1–10 *μ*M were selected. Four TCM injections, DHI (Buchang Pharmaceutical Co., Ltd., Shandong, China, batch number 12081024077), XDI (Bicon Pharmaceutical Co., Ltd., Jiangsu, China, batch number 120812), MLNI (Jinling Pharmaceutical Co., Ltd., Jiangsu, China, batch number 20121208), and FFKSI (Zhendong Pharmaceutical Co., Ltd., Shanxi, China, batch number 20130228), were purchased from the First Teaching Hospital of Tianjin University of Traditional Chinese Medicine. The TCM injections were serially diluted up to 1000-fold by DMEM/high glucose supplemented with 10% FBS. HepG2 cell line was purchased from the Type Culture Collection of the Chinese Academy of Sciences (Shanghai, China). The Mitotracker Deep Red FM fluorescent probe was obtained from Invitrogen (CA, USA), while other fluorescent probes were obtained from Sigma-Aldrich (MO, USA).

### 2.2. Cell Culture and Drug Treatment

HepG2 cells were subcultured less than fifteen generations after acquisition from the culture collection. Cells were cultured in DMEM/high glucose with 4500 mg·L^−1^ glucose and 4 mM·L^−1^-glutamine (Thermo Fisher Scientific, UT, USA) and supplemented with 10% FBS (Gibco, NY, USA) and 100 IU·mL^−1^ penicillin-streptomycin (Gibco, NY, USA) at 37°C in a 5% CO_2_ atmosphere. Cells were subcultured following trypsinization with a 0.25% trypsin-EDTA solution (Gibco, NY, USA), plated onto a Collagen I (Shengyou, Hangzhou, China) coated 96-well microplate (Corning, MA, USA) at a density of 8 × 10^3^ per well, and cultured at 37°C with 5% CO_2_. Only the inner 60 wells of 96-well microplate were used due to evaporation-related edge effects in the outside wells.

### 2.3. HCA Imaging and Image Analysis

In the assay, cell parameters associated with CN, NA (Hoechst 33342), MS (Mitotracker Deep Red FM), MMP (Rhodamine 123), and PMP (PI iodide) were measured. After 24-hour incubation, 50 *μ*L of a fluorophore mixture containing 3 *μ*mol·L^−1^ Hoechst 33342, 0.9 *μ*mol·L^−1^ Mitotracker Deep Red FM, and 9 *μ*mol·L^−1^ PI iodide in DMEM/high glucose was added to each well. Cells were incubated 30 minutes in dark. After removing the medium, 100 *μ*L 1.2 *μ*g·mL^−1^ Rhodamine 123 was added in each well. The cells were cultured for additional half hour prevented from light, and then they were washed once with warm DMEM before image scan. The assay plate was imaged and analyzed using the Operetta HCA system (Perkin Elmer, MA, USA) at 25°C with a relative humidity of 45% using a 20x objective. The fluorescent images of six fields per well in Hoechst 33342, Mitotracker Deep Red FM, PI iodide, and Rhodamine 123 channels were acquired by confocal scan, respectively. CN, NA, MS, MMP, and PMP were measured and calculated by the mean values of six images by adopting Graph Pad Grism (Graph Pad Software, Inc. CA, USA) software.

### 2.4. Subacute Toxicity Test

Animal care and operation procedures were in strict accordance with the China Laboratory Animal Use Regulations, and the animals were performed on in accordance with the institutional ethical guideline, and the experiment was approved by the institutional Animal Care and Use Committee of Tianjin International Joint Academy of Biotechnology and Medicine. Sprague-Dawley rats (male, 200 ± 20 g) were purchased from the Laboratory Animal Center of Academy of Military Medical Sciences and housed in stainless steel wire bottom cages with a control environment (25 ± 1°C, 50–60% humidity, 12 h fluorescent lighting per day) for 10 days of acclimatization. All rats were fed with standard food and water* ad libitum*.

Rats were randomly divided into nine groups of six animals each. 1.2 times of clinical dose was set as low-dose group. Four low-dose groups were administrated 4.4 mL·kg^−1^, 2.2 mL·kg^−1^, 1.3 mL·kg^−1^, and 2.2 mL·kg^−1^ of DHI, XDI, FFKSI, and MLNI, respectively while the high-dose groups were given 6 times of clinical dose of each injection. Control group received 22 mL·kg^−1^ physiological saline. All agents were administered daily for 28 days via tail vein injection. Body weights were recorded on the first day of the experiment and weekly thereafter. The dosage was corrected according to the weight change. Physical condition of animals was observed daily including changes in skin and fur, eyes and mucous membranes, and manure and behavior patterns. The rat sacrificed during the experiment was necropsied immediately; all macroscopic abnormalities of heart, liver, spleen, lung, kidney, and other organs were recorded. Since the intense adverse reactions of rats in FFKSI high-dose group, the rats were necropsied, and all organs and tissues were routinely processed on the 14th day.

### 2.5. Serum Biochemical Assays

After 28 days of drug treatment, rats were fasted overnight and blood samples were obtained from the abdominal aorta following anesthesia with 10% chloral hydrate (5 mL·kg^−1^) and plated at 4°C to clot for five hours. Serum was separated by centrifugation at 3000 r for 15 min. Biochemical parameters of serum enzyme activities of alanine aminotransferase (ALT), aspartate aminotransferase (AST), triglyceride (TG), and alkaline phosphatase (ALP) were measured by commercial ALT reagent kit (Biosino, Beijing, China), AST reagent kit (Biosino, Beijing, China), GPO-PAP TG kit (Biosino, Beijing, China), and ALP kit (Biosino, Beijing, China) using a Hitachi 7020 Clinical Chemistry Analyzer (HITACHI, Tokyo, Japan).

### 2.6. Antioxidative Effects

The livers of the rats were isolated immediately and washed in ice-cold physiological saline. 0.2 g liver tissue of each rat was weighted precisely and homogenized with physiological saline in a Teflon homogenizer and centrifuged at 3000 r for 10 min to get 10% liver homogenates (w/v). The activity of superoxide dismutase (SOD), catalase (CAT), glutathione (GSH), and malondialdehyde (MDA) was determined by using commercially SOD WST-kit (Jiancheng, Nanjing, China), CAT assay kit (Jiancheng, Nanjing, China), GSH assay kit (Jiancheng, Nanjing, China), and MDA assay kit (Jiancheng, Nanjing, China).

### 2.7. Statistical Analysis

IC 50 of six image fields per well was calculated by Graph Pad Grism software. All data was expressed as mean ± SEM. Statistical analysis was performed using ANOVA with LSD test by SPSS 17.0. Value of *P* < 0.05 was considered statistically significant.

## 3. Results

### 3.1. Method Validation with Positive Control Drugs

**Figure 1:**
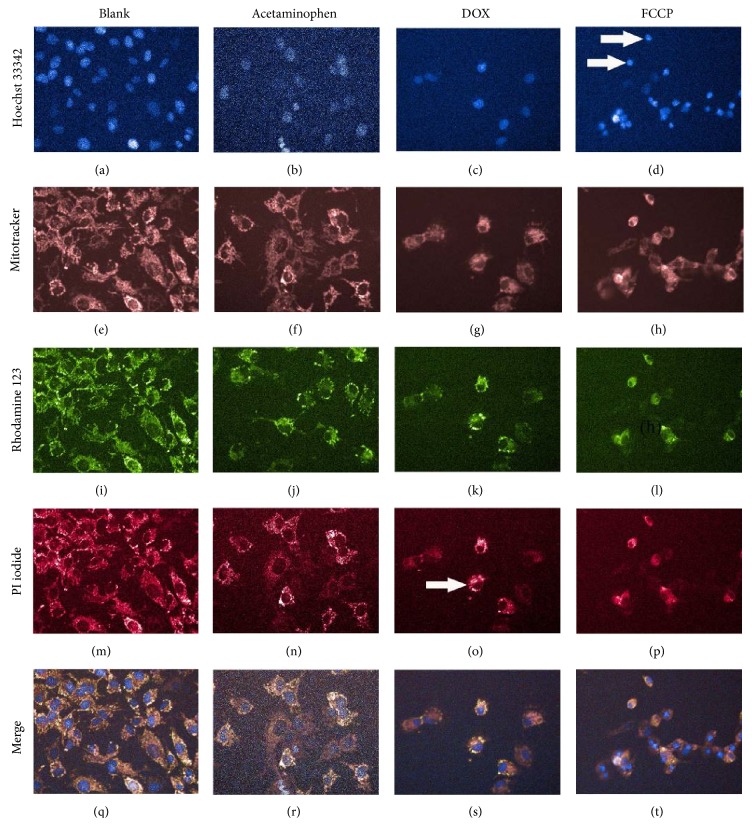
Representative images of HCA for positive drugs induced hepatotoxicity on Hoescht 33342, Mitotracker Deep Red FM, PI iodide, and Rhodamine 123 channels. HepG2 cells treated with blank (a, e, i, m, and q), 3 *μ*M FCCP (d, h, l, p, and t), 3 mM acetaminophen (b, f, j, n, and r), and 3 *μ*M doxorubicin hydrochloride (DOX) (c, g, k, o, and s) are shown. The increased intensity of Mitotracker Deep Red FM indicated an increased mitochondrial mass (MS) (g, h). The decreased intensity of Rhodamine 123 indicated a decreased mitochondrial membrane potential (MMP) (j, k, and l). The MMP decrease was indicated by white arrow (g, k, and o). The intensity of PI was increased when plasma membrane permeability (PMP) increased. The PMP increase was indicated by white arrow (o). The cell nucleuses stained by Hoechst 33342 shrunk and CN decreased by FCCP, indicated by white arrow (d).

**Figure 2:**
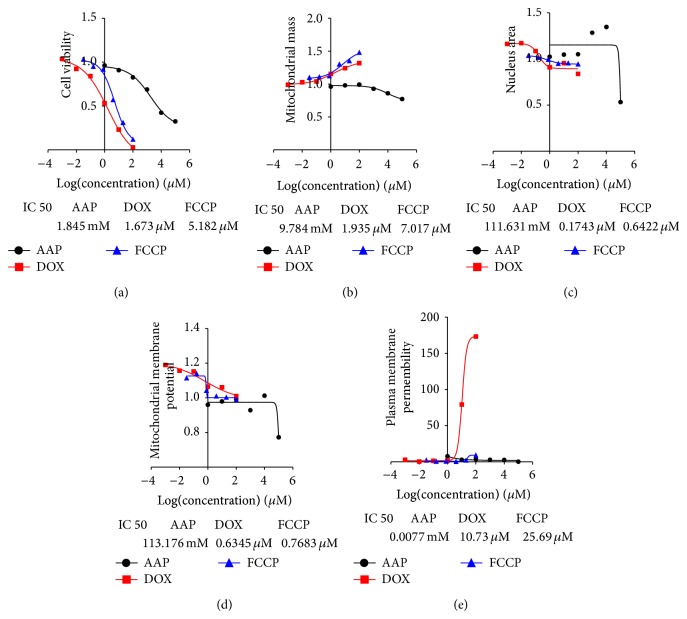
Acetaminophen (AAP), doxorubicin hydrochloride (DOX), and FCCP-generated dose-response curves deduced from cell number (a), mitochondrial mass (b), nuclear area (c), mitochondrial membrane potential (d), and plasma membrane permeability (e). Exposed to acetaminophen (1 *μ*M, 10 *μ*M, 100 *μ*M, 1 mM, 10 mM, and 100 mM), exposed to doxorubicin hydrochloride (1 nM, 10 nM, 100 nM, 1 *μ*M, 10 *μ*M, and 100 *μ*M), and exposed to FCCP (32 nM, 160 nM, 800 nM, 4 *μ*M, 20 *μ*M, and 100 *μ*M), HepG2 cells are studied. Data is expressed as mean ± SEM, *n* = 3.

The sensitivity of the multiparametric HCA assay was first validated by three known hepatotoxic compounds. Representative images of Hoescht 33342, Mitotracker Deep Red FM, PI iodide, and Rhodamine 123 channels captured by the HCA established typical cytotoxic effects caused by FCCP (3 *μ*M), acetaminophen (3 mM), and doxorubicin hydrochloride (3 *μ*M). As shown in Figure 1, an increased intensity of Mitotracker Deep Red FM indicated MS increase caused by FCCP and doxorubicin hydrochloride (Figures 1(g) and 1(h)); a decreased intensity of Rhodamine 123 indicated the MMP decrease caused by all three positive drugs (Figures 1(j), 1(k), and 1(l)). The cell nucleuses shrunk were founded by FCCP, Figure 1(d). As shown in Figure 2, FCCP at 3 *μ*M, 10 *μ*M, and 30 *μ*M (*P* < 0.01), acetaminophen at 1 mM, 3 mM, and 10 mM (*P* < 0.01), and doxorubicin hydrochloride at 0.1 *μ*M to 10 *μ*M (*P* < 0.01) dramatically decreased the CN by 35.8%–70.4% compared with blank group. FCCP at 30 *μ*M (*P* < 0.05), acetaminophen at 100 *μ*M (*P* < 0.01), and doxorubicin hydrochloride at 3 *μ*M and 10 *μ*M (*P* < 0.01) significantly increased the PMP by 17.5-, 61.4-, 19.0-, and 44.9-fold, respectively. Acetaminophen significantly increased the NA by 25.7% at 3 mM (*P* < 0.01), while it increased the MMP by 26.7% at 100 *μ*M (*P* < 0.01). In addition, for FCCP, acetaminophen, and doxorubicin hydrochloride, the IC 50 of CN was 5.18 *μ*M, 1.84 mM, and 1.67 *μ*M, respectively. For NA, the IC 50 was 0.60 *μ*M, 111.63 mM, and 0.17 *μ*M, respectively. For MS, the IC 50 was 7.02 *μ*M, 9.78 mM, and 1.94 *μ*M, respectively. For MMP, the IC 50 was 0.77 *μ*M, 113.18 mM, and 0.63 *μ*M, respectively. For FCCP, acetaminophen and doxorubicin hydrochloride, the IC 50 of PMP was 25.69 *μ*M, 0.01 mM, and 10.73 *μ*M.

### 3.2. Effect of TCM Injections in HCA Hepatotoxicity Assay

**Figure 3:**
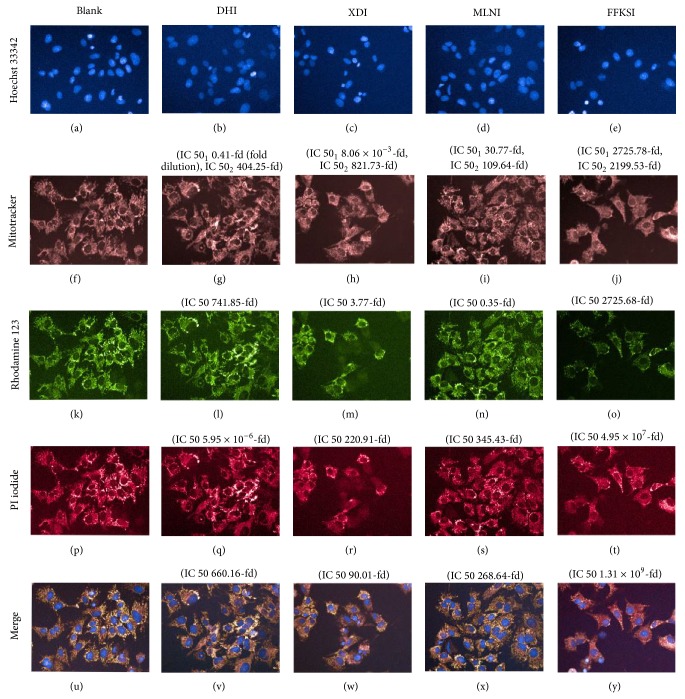
Representative images of the HCA analysis of HepG2 cells for the evaluation of nuclei number and morphology stained by 4 dyes in control (a, f, k, p, and u), Danhong injection (DHI) 100-fold dilution (b, g, l, q, and v), Xiangdan injection (XDI) 100-fold dilution (c, h, m, r, and w), Mailuoning injection (MLNI) 100-fold dilution (d, i, n, s, anad x), and Fufangkushen injection (FFKSI) 100-fold dilution (e, j, o, t, and y) groups. The cell number (CN) decreased by XDI 100-fold dilution and FFKSI 100-fold dilution (c, e). The intensity of Rhodamine 123 (green) was lower in FFKSI-treated group than control group indicating that the MMP decreased (o). Note: IC 50 of cell parameters associated with cell number (CN) was IC 50_1_ and IC 50 of cell parameters associated with nuclear area (NA) was IC 50_2_.

**Figure 4:**
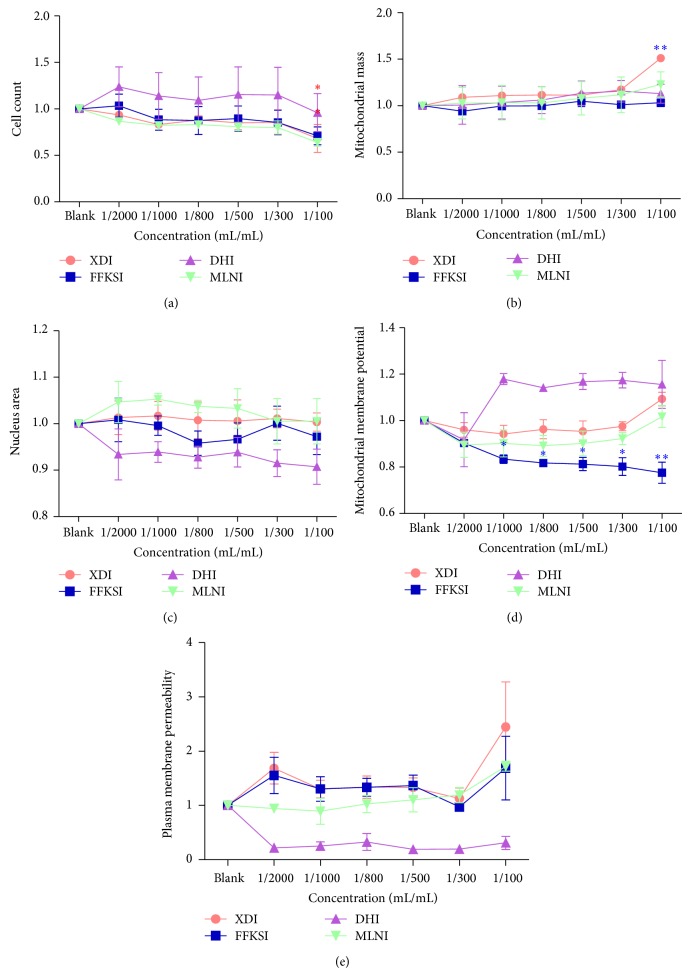
Effects of Danhong injection (DHI), Xiangdan injection (XDI), Mailuoning injection (MLNI), and Fufangkushen injection (FFKSI) on cell number (a), nuclear area (b), mitochondrial mass (c), mitochondrial membrane potential (d), and plasma membrane permeability (e). HepG2 cells were treated with DHI, XDI, MLNI, and FFKSI. FFKSI (100-fold dilution) and XDI (100-fold dilution) caused deviations from the control group with a significant decrease of the cell number (a). A significant increase of MS was induced by XDI (100-fold dilution) (b). All FFKSI group (100-, 300-, 500-, 800-, and 1000-fold dilution) induced a decrease of MMP (d). Data is expressed as mean ± SEM. ^*^ *P* < 0.05, ^**^ *P* < 0.01, *n* = 3.

Five fixed concentrations (1000-, 800-, 500-, 300-, 100-fold dilution) of four injections, DHI, XDI, FFKSI, and MLNI were selected. As shown in Figure 3, the representative images were captured by the HCA method established for typical cytotoxic effects on hepatocyte induced by FFKSI 100-fold dilution, XDI 100-fold dilution, MLNI 100-fold dilution, and DHI 100-fold dilution. It is noteworthy that 1000-, 800-, 500-, 300- (*P* < 0.05), and 100-fold dilution (*P* < 0.01) of FFKSI significantly decreased the MMP by 11.1%–22.8% as compared to blank group (Figure 4(d)). XDI and FFKSI at 100-fold dilution caused a significant decrease of CN by 50.8% and 48.4% (*P* < 0.05), respectively (Figure 4(a)). XDI at 100-fold dilution also showed a significant increase of MS (3237.24 ± 78.38) by 34.8% compared with blank group (2265.40 ± 109.58) (*P* < 0.01) (Figure 4(b)). As shown in Figure 4, DHI and MLNI at all concentrations did not have significant changes in any of the parameters studied. The result of HCA assay suggested that the high concentration of FFKSI and XDI may cause mitochondrial damage of HepG2 cells. In addition, for DHI, XDI, MLNI, and FFKSI, the IC 50 of CN was 0.41-fold, 8.6 × 10^−3^-fold, 30.77-fold, and 2725.78-fold; the IC 50 of MS was 741.85-fold, 3.77-fold, 0.35-fold, and 2725.68-fold; the IC 50 of NA was 404.25-fold, 821.73-fold, 109.64-fold, and 2199.53-fold; the IC 50 of MMP was 5.95 × 10^−6^-fold, 220.91-fold, 345.43-fold, and 4.95 × 10^7^-fold; the IC 50 of PMP was 660.16-fold, 90.01-fold, 268.64-fold, and 1.31 × 10^9^-fold.

### 3.3. Effect of TCM Injections in Subacute Toxicity Test

To confirm the HCA results, a subacute animal toxicity test was carried out. Of all 54 animals subjected to the subacute toxicity test, four were sacrificed during the experiment, two from FFKSI high-dose group, one from FFKSI low-dose group, and one from XDI high-dose group (Table 1). For the animals in the high-dose FFKSI group, twitching was observed in six rats immediately after drug injection and sustained nearly 3 minutes. Since intense adverse reactions occurred in the group with high-dose FFKSI, the rats were necropsied and all organs and tissues were routinely processed on the 14th day. Body weight of rats in FFKSI low-dose group increased slowly. On the 7th day after injection, the rats were irritable and the fur became pale brown. Lower limbs of one rat were paralyzed after drug administration on the 23th day and the rat died 2 days later. The body weight of rats in XDI high-dose group increased slowly and the fur was lackluster. One rat lower limbs were paralyzed on the 17th day after injection and the rat died 3 days later.

**Figure 5:**
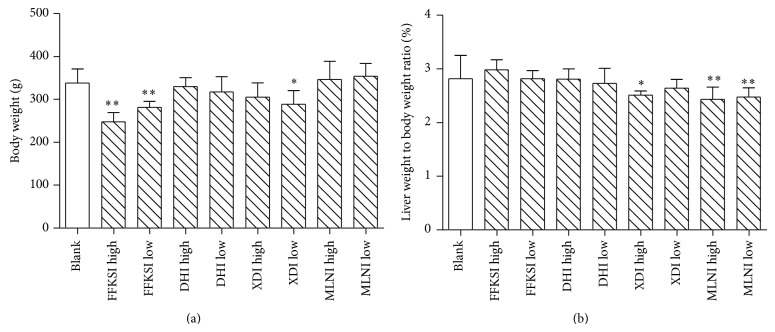
The body weight (a) and the ratio of liver weight to body weight (b) of rats after 28-day injection of Danhong injection, Xiangdan injection, Mailuoning injection, and Fufangkushen injection. Data are given as mean ± SEM, ^*^ *P* < 0.05, ^**^ *P* < 0.01.

The effects of four injections on the growth of rats are shown in Figure 5. The body weight decreased significantly in both FFKSI high-dose and low-dose group (*P* < 0.01) as well as in XDI low-dose group (*P* < 0.05) by 16.9%, 26.8%, and 16.0%, respectively (Figure 5(a)). DHI and MLNI did not change body weight after 28 days of injection. XDI in high-dose (*P* < 0.05) as well as MLNI in both low-dose and high-dose groups (*P* < 0.01) decreased the ratio of liver weight to body weight significantly compared to those in control group (Figure 5(b)), while DHI and FFKSI did not change it.

### 3.4. Effect of 4 TCM Injections by Serum Biochemistry Analysis

After 28 days of administration of four injections, serum activities of AST, ALT, TG, and ALP enzymes in all groups did not change significantly compared to control group. No influence on liver function caused by 4 TCM injections was observed from the serum biochemical study.

### 3.5. Effect of TCM Injections on Hepatic Antioxidant Enzyme Activities and Lipid Peroxidation

**Figure 6:**
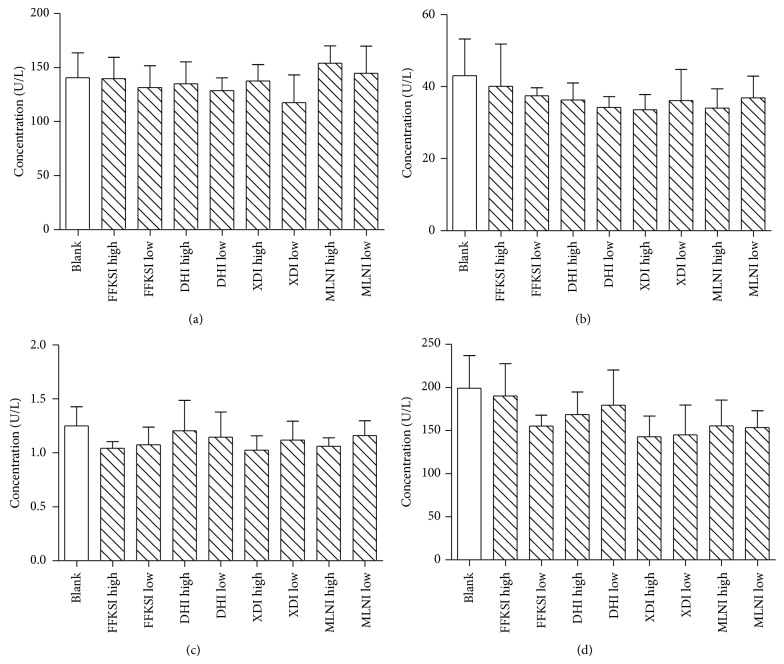
Serum AST (a), ALT (b), TG (c), and ALP (d) profile of Danhong injection, Xiangdan injection, Mailuoning injection, and Fufangkushen injection after 4-week treatment to rats. Data are given as mean ± SEM for each point of at least four separate rats for each point, ^*^ *P* < 0.05, ^**^ *P* < 0.01.

**Figure 7:**
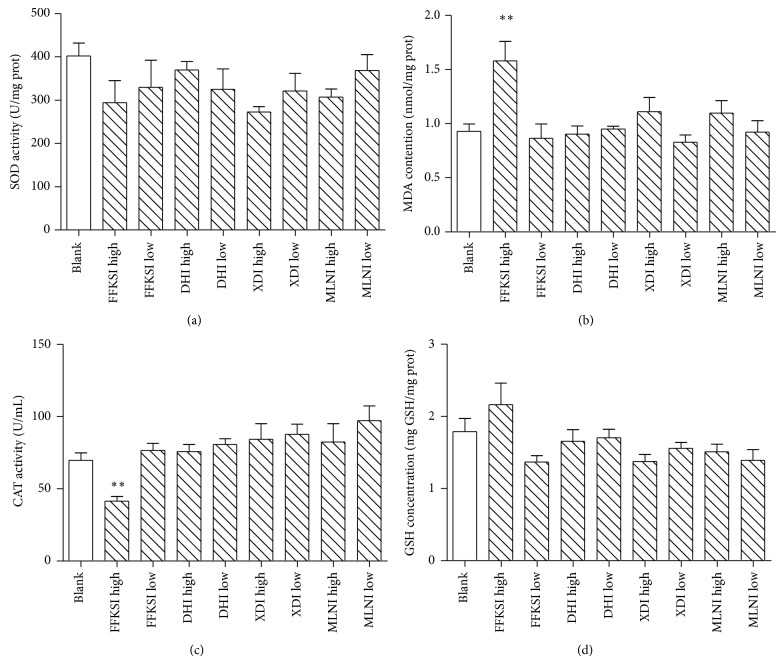
The effect of Danhong injection, Xiangdan injection, Mailuoning injection, and Fufangkushen injection on SOD (a), MDA (b), CAT (c), and GSH (d) in liver homogenate. In FFKSI high-dose group, the CAT activity decreased extremely significantly (b) and MDA concentration increased significantly (c). Data are given as mean ± SEM, ^*^ *P* < 0.05, ^**^ *P* < 0.01.

To further assess the antioxidant effect of the four injections on liver function* in vivo*, SOD, MDA, CAT, and GSH were measured. As shown in Figure 6, the SOD, MDA, CAT, and GSH activities of 4 TCM injections did not show significant change except for the high-dose group of FFKSI. The CAT activity of FFKSI high-dose group (41.61 ± 3.11, *P* < 0.01) decreased 40.4% compared to the blank; meanwhile the MDA activity (1.57 ± 0.18, *P* < 0.01) increased significantly by 70.0%. It indicated that the high dose of FFKSI increased reactive oxygen species (ROS) and decreased the antioxidant capacity of liver tissue that was in accordance with the decrease of MMP found in HCA assay established. The SOD value of XDI high-dose group was lower than all other groups; the MDA level was also higher than other groups except for FFKSI high-dose group without significant difference. It indicated that long-term use of XDI at a high concentration may also cause certain oxidative damage on liver (Figure 7).

## 4. Discussion

With increased application of TCM injections in clinical practice, safety problems have been increasing. The four TCM injections investigated in this study were widely used in clinical practice in China with large patient population. In 2013, the sales amount of the four injections totaled to more than 4 billion RMB. Different degrees of hepatotoxicity ADR reports of the four TCM injections were collected from clinical centers and SFDA reports. XDI and MLNI were reported in ADR information bulletin [8, 9]. FFKSI increased ALT, AST, and ALP [7]; XDI increased ALT, chronic hepatitis B, and liver cirrhosis in clinical treatment [10, 11]. MLNI increased ALT and AST at 68–229 U/L and 30–157 U/L after 2 weeks of use [12, 13]. The incidence of ADR by DHI is relatively low (5.15‰ ) [14] and its hepatotoxicity adverse reaction has rarely been recorded. Therefore, a rapid and accurate method is needed to postmarketing reevaluation of hepatotoxicity of TCM injection.

Cell-based assays have been increasingly employed in predicting drug-induced toxicities [23], which is an efficient means to reduce the dependence on animals. Multiparameter HCA assays have so far been applied to measure genotoxicity, hepatotoxicity, drug-induced phospholipidosis, and developmental neurotoxicity. In terms of hepatotoxicity, O'Brien et al. [5] applied HCA and tested 243 known hepatotoxicity drugs using HepG2 human hepatocytes with a specificity of 98% and a sensitivity of 93%. Based on O'Brien's work, a live cell multiparametric HCA cytotoxicity assay was developed by Abraham et al. [24]. Seventeen compounds with various hepatotoxicity mechanisms were evaluated. The capability of HCA assay on sensitively detection with different mechanism was approved. Garside et al. [25] developed a HCA hepatotoxicity assay on HepG2 human hepatocytes and 144 compounds were assessed. The specificity and sensitivity were 58% and 75%, respectively. However, HCA-based hepatotoxicity assay has not been attempted for evaluating either drug combinations or TCM or herbal medicine that have complex chemical constituents and potential multiple toxicity determinants. Due to its complex chemical nature, safety evaluation of TCM preparations has been a challenging task, especially on providing an organ-specific toxicity profile and a mechanistic insight. The ability to determine multiple cell parameters associated with cell health by the application of microfluorescent, multiprobe technology simultaneously makes HCA a suitable tool for safety assessment of TCM preparations with complex chemicals. Compared to conventional assays, the higher sensitivity of HCA assay can provide clear image of cells and quantify multiple cellular biomarkers to evaluate the effect of compound and analysis the pathogenesis of toxicity. Long term and acute toxicity test on various animal species is necessary for new drug approval. However, unpredictable ADR still cannot be avoided according to the postmarket surveillance. The HCA assay established on human cells is highly predictive for drug-induced liver injury. It can be employed in drug discovery and postmarket reevaluation to optimize compound series on their safety profile and provide a risk assessment tool towards candidate selection with higher output and sensitivity.

The median concentrations of FCCP and acetaminophen referred to IC 50 from the assay protocol of Operetta HCA system were 5.2 *μ*M and 1.3 mM. The lowest concentration of doxorubicin hydrochloride was set as 0.1 *μ*M according to previous research [4]. In order to confirm dose-response relationship, FCCP (0.32–100 *μ*M), acetaminophen (0.001–100 mM), and doxorubicin hydrochloride (0.001–100 *μ*M) were selected. Since there is no sufficient evidence of relationship between the amount used in* in vitro* cell experiment and clinical dose of TCM injections, the formulation was chosen to calculate the highest concentration of injections in the HCA assay. Consider the following:

(1) Highest  concentration  of  TCM  injection =60×D/500050%,

where 60 is average weight of adult, *D* is the clinic dosage of TCM injections (mL·kg^−1^d^−1^), 5000 is average blood volume (mL) of adult, and 50% is the volume ratio of blood cells in plasma. The clinic dosage of DHI is 0.67 mL·kg^−1^d^−1^; for instance, 100-fold diluted DHI was calculated and set as the highest concentration [26].

In the subacute animal test, according to “Guide on New Drug research of Chinese Medicine” [27], 1.2 times of the clinical dose was set as low-dose group with administration amount of 4.4 mL·kg^−1^, 2.2 mL·kg^−1^, 1.3 mL·kg^−1^, and 2.2 mL·kg^−1^ of DHI, XDI, FFKSI, and MLNI, respectively. Meanwhile 6 times of clinical dose was the high-dose group.

Given the need for a proliferating cell model for predictive cytotoxicity studies, the effectiveness of the choice of HepG2 human cells as a cell line to drug safety evaluation is supported by other studies. Schoonen et al. found them slightly more predictive than HeLa, ECC-1, and CHO-k1cells [15, 16]. Others have made similar findings (Bugelski et al. 2000) [17]. A cell line with metabolic competence (HepaRG cells) and morphology more representative of the* in vivo* state (L-02 cells) would likely further enhance the predictability of cytotoxicity assays in our following study.

In this study, five parameters (CN, MS, MMP, NA, and PMP) were simultaneously assessed to evaluate cell proliferation, mitochondrial function, and cellular structure integrity. Cell permeable dye Hoechst 33342 was used to locate the cell and measure NM and CN. CN is one of the most sensitive cell health indicators directly affected by drug toxicity, while NA change indicates the damage of nucleus, alive cell impermeable DNA binding dye, PI iodide, measures PMP via quantification of nuclear brightness.

Mitochondria is one of the most important target sites 14 of hepatotoxicity drugs. According to the three-step model of drug induced liver injury, the decrease of MMP is the most important step in the process [28]. When the mitochondrial function was impaired, the MMP would decrease and cytochrome C and other apoptosis inducing factors would be released from mitochondria into cytosol to trigger subsequent activation of procaspase-9 and downstream apoptotic effectors to cause apoptosis [6, 29]. In our assay, Mitotracker Deep Red FM and Rhodamine 123 were chosen to measure mitochondrial function. Rhodamine 123 accumulates in mitochondria depending on MMP whereas Mitotracker Deep Red FM stains mitochondria without the influence from MMP. The combined application enhanced the accuracy in deeply damaged mitochondria. Since mitochondria is the main target in the process of liver injury and insensitivity of intracellular calcium, mitochondrial mass was adopted as a substitution of Ca2+ membrane permeability in the HCA assay established by O'Brien et al. [5]. It is appropriate for hepatotoxicity evaluation of the natural product with complex chemical constituents.

In the subacute animal test, 4 rats were sacrificed because of faulty operation on injection rate and infusing air, two from FFKSI high-dose group, one from FFKSI low-dose group, and one from XDI high-dose group. The rats were fiercely struggled when injecting FFKSI; twitching was observed immediately after injection and sustained nearly 3 minutes on six rats and 2 rats died in the process of injecting in high-dose group. No more rats died when injection speed was turned down. In the FFKSI low-dose group, the body weight of rats increased slowly, and on the 7th day after injection, the rats were irritable and the fur became pale brown. One rat lower limbs were paralyzed after administrated on the 23th day after injection and the rat died 2 days later.

Serum concentrations of AST, ALT, ALP, and TG are important indicators of hepatic health [30, 31]. The activity of AST, ALT, and TG enzymes in all groups of the four injections showed no significant difference compared with control after 28 days of injection. It indicated that the 4 TCM injections would not induce severe histopathological hepatic lesions in rats.

Excessive free radicals would consume intracellular SOD, CAT, and GSH and cause hepatocyte damage in liver cells [32]. MDA is an end product of lipid peroxidation. The concentration of MDA is widely used as a marker of free radical mediated lipid peroxidation injury of liver tissue [33]. According to biochemical experiment of FFKSI high-dose group, cell antioxidant activity weakened along with the increased MDA content and decrease of CAT activity in liver homogenate, indicating that the high-dose FFKSI weakened the antioxidant capacity of liver cells. A concentration dependent decrease of CN and MMP was found from 1000- to 100-fold dilution of FFKSI by the HCA assay established, indicating that FFKSI damaged the cells by inducing mitochondrial dysfunction. The hepatotoxicity caused by FFKSI was probably relative with the damage of liver cells by reducing the antioxidant ability, promoting the formation of ROS and damaging the mitochondrial membrane structure.

The 100-fold dilution of XDI caused significant decrease of CN and significant increase of MS from the HCA result. In subacute toxicity test, one rat in XDI high-dose group was sacrificed during the experiment. The SOD value of XDI was lower than other groups while the MDA level was higher than all other groups except for FFKSI high-dose group, though there was no significant difference among those groups. In addition, the body weight in XDI low-dose group and the ratio of liver weight to body weight in XDI high-dose group are significantly lower than blank. It indicated that long-term use of XDI at a high concentration may also cause certain oxidative damage on liver by affecting the function of mitochondria. High correlation was found between the results of HCA hepatotoxicity assay and the subacute animal test.

In summary, we present an* in vitro *cell-based high-content assay using PerkinElmer HCA system, which is highly predictive for human drug-induced liver injury. The hepatotoxicity of four TCM injections was tested. The result is in accordance with subacute animal study. Compared with conventional hepatotoxicity assays, the assay is sensitive and accurate with higher throughput, lower cost, and less animals needed that is suitable for TCM preparations with complex chemicals.

## Figures and Tables

**Table 1:** Phenotypic changes in subacute toxicity test.

Group	Death/total animals	Abnormalities
Control	0/6	Normal
DHI high-dose	0/6	Normal
DHI low-dose	0/6	Normal
XDI high-dose	1/6	1/6 hind limb paralyzed
XDI low-dose	0/6	Normal
MLNI high-dose	0/6	Normal
MLNI low-dose	0/6	Normal
FFKSI high-dose	2/6	6/6 twitched after injection
FFKSI low-dose	1/6	1/6 hind limb paralyzed
